# Reproducibility of a nylon fishing line as a screening test for diabetic foot ulceration risk

**DOI:** 10.6061/clinics/2020/e1658

**Published:** 2020-04-20

**Authors:** Mozânia Reis de Matos, Daniele Pereira Santos‐Bezerra, Maria Lucia Correa-Giannella

**Affiliations:** I Programa de Pos-Graduacao em Medicina, Universidade Nove de Julho (UNINOVE), Sao Paulo, SP, BR; II Unidade Basica de Saude Dra Ilza Weltman Hutzler, Sao Paulo, SP, BR; III Laboratorio de Carboidratos e Radioimunoensaio (LIM-18), Hospital das Clinicas HCFMUSP, Faculdade de Medicina, Universidade de Sao Paulo, Sao Paulo, SP, BR

The reproducibility of using the “homemade” monofilament (a nylon fishing line) proposed by Parisi et al. in the manuscript titled “Diabetic foot screening: Study of a 3000 times cheaper instrument” 1 was tested in this study. Independent validation was performed in a primary care setting, a basic health unit located on the outskirts of the city of São Paulo, which is responsible for the care of approximately 2,000 individuals with diabetes mellitus. A total of 548 out of 853 individuals with type 2 diabetes invited to participate in the study was included (59.3% women; median [interquartile interval] age, 65 [59–72] years; median diabetes duration, 10 [5–15] years; median HbA1c, 7.2% [6.3%–9.1%]). The study complied with the Declaration of Helsinki and received approval from the institutional ethics committee. All participants provided written informed consent. The performance of Semmes Weinstein's 10-g monofilament (Sorri Bauru®, São Paulo, Brazil), considered the gold standard for detecting ulcer risk 2, was compared to that of the nylon fishing line (Nylon 6 [homopolymer]; diameter, 0.50 mm; length, 4 cm; Trevo, Equipesca, São Paulo, Brazil) 1 at the three recommended sites (hallux and first and fifth metatarsal heads) 3. The kappa coefficient, used as a measure of inter-annotator agreement for categorical items, was expected to be 1 if the two instruments were in complete agreement. As shown in Table 1, the correlation coefficients corroborated the equivalence of the nylon fishing line with Semmes Weinstein's 10g monofilament. This finding reinforces that the fishing line is an effective alternative to the commercially available 10g monofilament as a tool for screening individuals with diabetes at risk for foot ulceration. Its low cost may enable its widespread use, especially in the primary care setting where resources may be limited, thus contributing to the reduction of foot ulcer occurrence and lower-limb amputations.

## Figures and Tables

**Table 1:** Correlation coefficients for the three sites tested with Semmes Weinstein’s monofilament and the nylon fishing line.

Right foot	Hallux	First metatarsal head	Fifth metatarsal head
Kappa	1.00	0.92	0.91
Standard error	0.00	0.05	0.05

Left foot	Hallux	First metatarsal head	Fifth metatarsal head
Kappa	1.00	0.92	0.96
Standard error	0.00	0.07	0.04
